# The Prevalence of Fabry Disease Among Young Cryptogenic Stroke Patients

**DOI:** 10.7759/cureus.9415

**Published:** 2020-07-27

**Authors:** Mohammed A Alhazzaa, Ahmed Mujtaba, Mohammed A Aljohani, Fatimah Alqarni, Roaa Alsharif

**Affiliations:** 1 Neurology, King Fahad Medical City, Riyadh, SAU; 2 Neurology, King Fahad Hospital, Madinah, SAU; 3 Research Center, King Fahad Medical City, Riyadh, SAU

**Keywords:** stroke, cryptogenic strokes, brain infarct

## Abstract

Introduction

Fabry disease is a metabolic storage disorder that causes disorders in multiple organs including the brain. Data regarding the prevalence of the disease among the Saudi stroke population is scarce. Hence, tests for the same are not conducted on a regular basis when investigating stroke of uncertain cause. Our study aimed to provide insight into whether testing for Fabry disease is justifiable in cryptogenic stroke patients who have no other features of the disease.

Method

This was a prospective study conducted at a single stroke center. We included young patients between the ages of 18 and 55 years who had confirmed and unexplained ischemic or hemorrhagic insults. Alpha-galactosidase enzyme testing was conducted in all suspected cases. Further genetic testing was performed in patients with abnormal enzyme results.

Result

A total of 51 patients met the inclusion criteria. The mean age was 42 years. All the included patients completed a workup of ischemia or hemorrhage. All cases had no clear etiology of their vascular events. All included patients lacked classic systemic manifestations of Fabry disease. We identified one case of borderline low α-galactosidase A (GLA) enzyme level. However, GLA genetic testing did not reveal any Fabry disease-related mutation. The study did not identify any subject with confirmed Fabry disease.

Conclusion

In this single-center study, we found that Fabry disease had a low prevalence among Saudi cryptogenic stroke patients who lack other systemic manifestations. Hence, Fabry testing is not generally considered in routine workup related to cryptogenic stroke.

## Introduction

Fabry disease is an X-linked inherited lysosomal storage disease, which is caused by a deficiency of α-galactosidase enzyme. Mutations in α-galactosidase A (GLA) gene lead to a reduction in enzyme activity. As a result, impaired glycosphingolipid metabolism results in glycosphingolipid accumulation, especially in vascular endothelial, cardiac, and renal cells [1]. Screening for Fabry disease can be reliably performed by GLA enzyme activity analysis; however, the detection of mutations in the GLA gene is the preferred method for diagnosis verification. Fabry disease generally starts in childhood and progresses into heart failure or renal failure in adulthood [1]. Ischemic stroke is a potential complication, which may occur later in the fourth or fifth decade of life [2]. Therefore, early recognition of Fabry disease is critical for favorable outcomes, and enzyme replacement therapy is available to treat the condition.

The Kingdom of Saudi Arabia (KSA) lacks comprehensive methodological studies specifically assessing the prevalence of this disease among adult cryptogenic stroke population. Hence, we sought to evaluate the prevalence of Fabry disease among Saudi citizens who had suffered an unexplained stroke. This may also provide insight into the value of testing for Fabry disease when no other clinical features of the disease are present.

## Materials and methods

This was a single-center study, conducted at a comprehensive stroke program within King Fahad Medical City in Riyadh, KSA. Between 2017 and 2019, we prospectively screened and collected data related to cases seen in the outpatient stroke prevention clinic or those admitted to the Acute Stroke Unit. Data collected included demographics, clinical presentations, and radiological information. We included young patients between 18-55 years of age from both genders who either had an unexplained transient ischemic attack (TIA), ischemic stroke, or intracerebral hemorrhage, confirmed by CT and/or MRI of the brain. We excluded all cases with incomplete standard workup or confirmed causes of strokes such as atrial fibrillation, valvular heart disease, carotid stenosis, hypercoagulable conditions, arterial dissection, or cardiac conditions with cardiac thrombus.

The blood samples were collected by a trained nurse and then sent for analysis. Sanofi Genzyme (Cambridge, MA) provided the investigators with dry blood spot (DBS) kits and covered the cost of GLA enzyme assay and gene analysis for all patients enrolled in this study. All blood samples were sent to an approved laboratory for GLA enzyme testing (Centogene GmbH, Rostock, Germany). When enzyme deficiency was suspected, the GLA gene was evaluated for targeted mutations by performing DNA sequencing of the chromosome Xq22.1.

Baseline and demographic characteristics were presented in frequencies and percentages, whereas all continuous variables were expressed as means. An extensive comparative analysis was not required. Our local Institutional Review Board (IRB) approved this protocol and its amendments. All included subjects in this study signed a consent form that described the study and provided enough information for subjects to make an informed decision about their participation in this study.

## Results

More than 500 patients were screened for the study, but only 51 patients met the inclusion criteria. The mean age was 42 years. The demographic features are presented in Table 1. All included patients were extensively investigated for any unusual underlying causes with brain imaging, echocardiography, thrombophilia tests, vasculitis workup, and heart rhythm monitoring. One or more of the vascular risk factors (including diabetes, hypertension, and dyslipidemia) were present in 30 patients. All cases had no clear etiology of their vascular events. All included patients did not have any other systemic manifestations of Fabry disease. All patients had DBS sent for analysis of the GLA enzyme. We identified one case of low enzyme level. This case was a female patient with posterior circulation ischemic stroke. She underwent GLA genetic testing, which did not reveal the typical target mutation for Fabry disease. The study failed to identify any subject with confirmed Fabry disease.

**Table 1:** Demographic data of the subjects

Variable	N	%
Male	31	60
Diabetes	20	40
Hypertension	24	47
Dyslipidemia	19	37
Transient ischemic attack	4	8
Ischemic stroke	43	84
Intracerebral hemorrhage	4	8
Posterior circulation stroke	13	30

## Discussion

This study did not detect a definite case of Fabry disease among the included subjects. We had one case of borderline low enzymatic level but genetically undetected mutation. Our finding may suggest a low prevalence of Fabry disease among cryptogenic stroke patients in our population. This has implications on the extent of any planned workup and whether it is worth testing for Fabry disease in stroke patients with unclear etiology. In light of this study, testing for Fabry disease may not be cost-effective among the cryptogenic stroke population in KSA.

The result of the present study is in keeping with other population-based studies where a similar issue was investigated. In a Canadian cohort of cryptogenic ischemic vascular events, the prevalence of Fabry disease was low, estimated to be around 0.3% [3,4]. Almost similar findings were reported in other studies among different populations [5,6,7]. This probably indicates homogeneous results among different populations despite variation in ethnic backgrounds and study-related methodologies. 

Our study has some limitations as it was conducted at a single tertiary center. We may have missed potential candidates due to referral bias as only selected patients are usually accepted at our center as per hospital regulations. We expected a higher number but faced slow recruitment due to restricted inclusion criteria and short study duration. In addition, an embolic stroke of undetermined source (ESUS) is not common, which added more challenges. Studies involving more centers with longer duration would help in arriving at results closer to reality. More than half of our cohort suffered regular vascular risk factors such as diabetes, hypertension, and/or dyslipidemia (Table 1). These factors might have contributed to the presenting strokes. We did not include cases of lacunar infarcts in order to reduce the influential effect on the study outcome. However, those diseases are prevalent among our population, and they may coincide with Fabry disease. In fact, excluding patients with such risk factors may lead to missing Fabry cases.

## Conclusions

Based on our findings, Fabry disease seems to have a low prevalence within the cryptogenic stroke population. Therefore, regular screening of Fabry disease may not be helpful in cases of unexplained vascular events, especially when other disease features are not present. There are still unanswered questions about unrecognized causes of ischemic stroke in young patients. This is a challenging issue worldwide, which has led to extensive loss of resources. Early identification of Fabry disease would help in implementing proper therapy and reduce its potential complications. However, it is still uncertain whether enzyme replacement would modify the risk of stroke or change the outcome after a vascular event. This should be an area for future research.
